# The mysterious route of sterols in oomycetes

**DOI:** 10.1371/journal.ppat.1009591

**Published:** 2021-06-17

**Authors:** Weizhen Wang, Xili Liu, Francine Govers

**Affiliations:** 1 Department of Plant Pathology, College of Plant Protection, China Agricultural University, Beijing, China; 2 Laboratory of Phytopathology, Wageningen University & Research, Wageningen, the Netherlands; 3 State Key Laboratory of Crop Stress Biology for Arid Areas, College of Plant Protection, Northwest A&F University, Yangling, China; THE SAINSBURY LABORATORY, UNITED KINGDOM

Sterols are a class of lipids with essential roles in sustaining the domain structure of cell membranes and regulating biological processes [1]. Human health is affected by high cholesterol levels [2]; drugs preventing this are widely used. Also striking is the high demand for compounds classified as sterol biosynthesis inhibitors (SBIs), either as medicine to control fungal infections or as agrochemicals to combat fungal plant diseases [3,4]. Despite the essential role of sterols in cell functioning in eukaryotes, several organisms, including nematodes, insects, and plasmodia, are not able to synthesize sterols themselves. To sustain normal development, these sterol auxotrophs may have to acquire exogenous sterols from their food or environment [5,6].

Here, we address questions related to sterol auxotrophy in oomycetes, organisms with a fungal-like morphology, but classified as Stramenopiles, together with among others, diatoms and numerous uncharacterized marine organisms [7]. Oomycetes are notorious as pathogens, mainly on plants, but also animals and other microbes can fall victim [8]. Best known is *Phytophthora*, a genus comprising over 150 described species that cause substantial damage in crop plants and forests. Although being sterol auxotroph, *Phytophthora* spp. can be cultured in vitro without sterols, but exogenously added sterols promote vegetative growth and reproduction [9,10]. It is therefore conceivable that oomycetes recruit sterols from their environment while invading their hosts. However, the exact mechanism of sterol recruitment is still unknown. Also, it is not clear to what extent sterols play a role in pathogen–host interactions, what happens with the sterols after recruitment, and how sterol signaling is mediated in oomycetes. Gathering the current knowledge may help solve these mysteries.

## Can oomycetes synthesize sterols?

**Fig 1:**
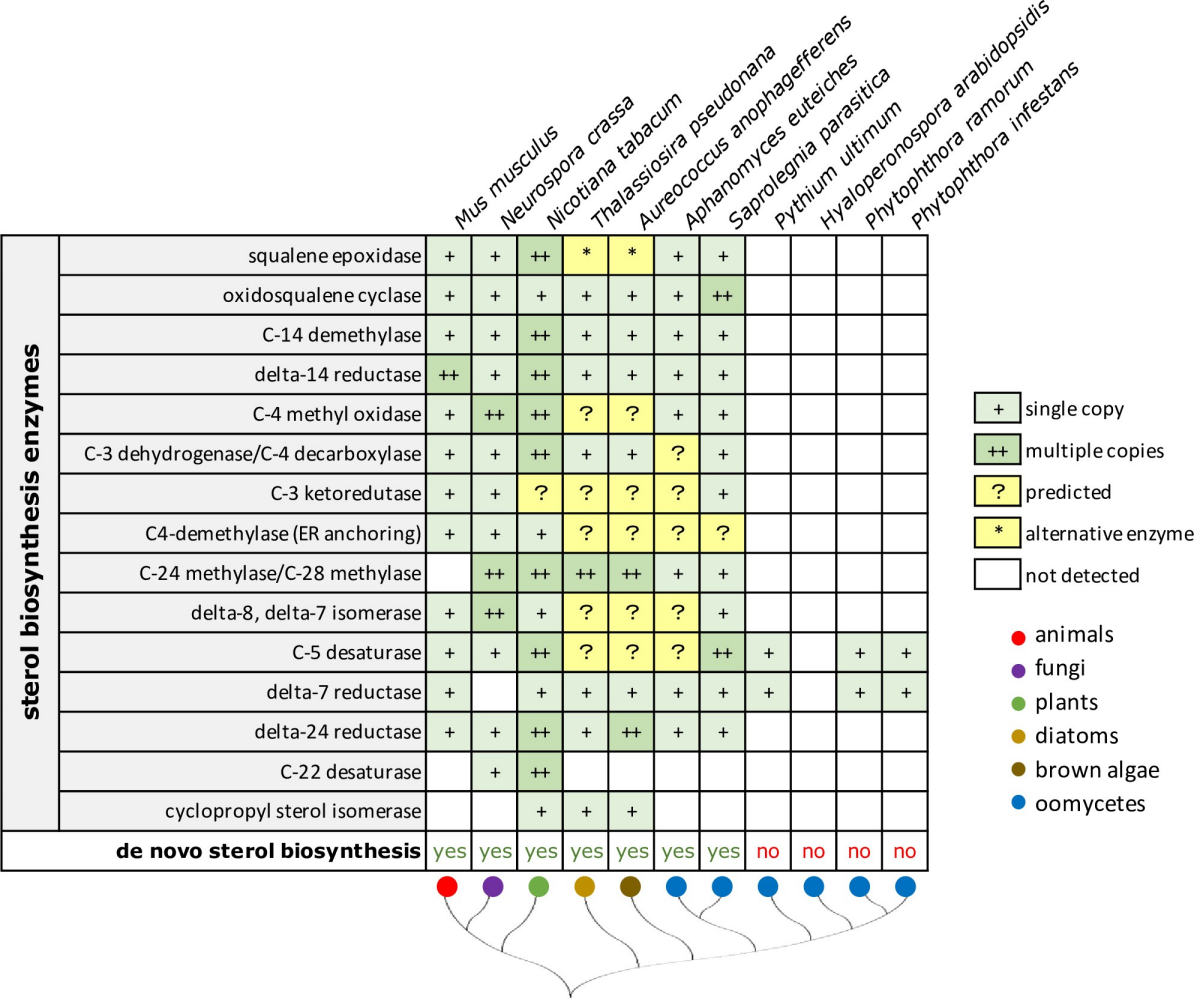
Sterol-auxotrophic oomycetes lack most canonical sterol biosynthesis enzymes. The sequential order of enzymes in the sterol biosynthesis pathway (left) and presence of the encoding genes in representative species from different eukaryotic lineages (in green). The simplified tree (bottom) shows the Stramenopiles in the right branch. It is anticipated that organisms showing de novo sterol biosynthesis have the complete set of enzymes. Most sterol autotrophs indeed have one (+) or multiple (++) copies of the encoding genes. However, not all genes that are predicted to be present have been detected (?). Possibly, there are alternative enzymes that take over, as is the case for squalene oxidase in some diatoms and brown algae (*). The sterol auxotrophs lack all or nearly all genes (▯). Data are from literature or obtained by mining databases.

Sterol biosynthesis is a multistep process catalyzed by a series of enzymes [11]. The universal precursor is the isoprenoid squalene, and biosynthesis starts with squalene oxygenation by squalene epoxidase followed by cyclization by oxidosqualene cyclase. Already in this step, the biosynthesis pathways start to diverge, resulting in different end products: cholesterol in animals, ergosterol in fungi, and campesterol and stigmasterol in land plants. Nevertheless, most enzymes in these diverging pathways are conserved (**Fig 1**) and rooted in the last eukaryotic common ancestor (LECA). Phylogenomic analyses show that the capacity to synthesize sterols evolved early during evolution and suggest that sterol auxotrophy resulted from independent gene losses in multiple lineages [12].

When emphasizing the differences between fungi and oomycetes, plant pathologists usually mention sterol auxotrophy as a typical characteristic of oomycetes, especially in connection with the efficacy of crop protection agents. Indeed, species belonging to the Perenosporales, a lineage mainly comprising plant pathogens, do not produce sterols. These pathogens are often highly adapted to their hosts; they have limited saprophytic capability and take advantage of sterols provided by the plant. In contrast, Saprolegniales species can produce sterols. In the legume pathogen *Aphanomyces euteiches*, for example, fucosterol was detected as the major sterol [13], while in the fish pathogen *Saprolegnia parasitica*, cholesterol derivatives are most prominent [14]. Saprolegniales are mostly opportunistic pathogens primarily growing as saprophytes apart from their hosts and thus cannot always rely on a sterol reservoir in their immediate surroundings. Moreover, also the more basal *Achlya* species should be able to produce sterols since their sex hormones are sterol derivatives [15]. So, in theory, these oomycetes should harbor an entire set of enzymes required for sterol biosynthesis (**Fig 1**), and, presumably, also the LECA of oomycetes had the capacity to synthesize sterols.

Interestingly, sterol auxotroph oomycetes still have remnants in their genomes reminiscent of this ancestral sterol biosynthesis pathway as evidenced by the preservation of homologs of *ERG3* and *DHCR7*. These genes, encoding C-5 sterol desaturase (EC 1.14.19.20) and Δ^7^-sterol reductase (EC 1.3.1.21), respectively, are still present in *Phytophthora* and *Pythium* but have been lost in downy mildews, obligate plant pathogens that entirely rely on their hosts for survival [12,16] (**Fig 1**). Why *ERG3* and *DHCR7* have been retained is unknown. Both enzymes function in the last steps of the pathway, and, possibly, they are used to modify sterol derivatives recruited from the environment or the diet. For DHCR7 in *Phytophthora capsici*, we have evidence that this is indeed the case. It can indeed convert ergosterol into brassicasterol, and this modification seems to be essential for the completion of the asexual life cycle under certain conditions [17]. For producing zoospores in vitro, *P*. *capsici* requires medium supplemented with sterols. CRISPR/Cas-9–mediated knock-out of *DHCR7* resulted in strains that no longer responded to ergosterol; unlike the wild-type strain, they could hardly form zoospores on medium supplemented with ergosterol [17].

## Can oomycetes survive without sterols?

Although sterols are deemed necessary for viability, *Phytophthora* and *Pythium* species can grow on sterol-free culture medium. However, as already reported in early studies [15], adding sterols to the medium not only stimulated their growth but also induced sporulation or initiated oospore formation in homothallic species. So, to reproduce and complete their life cycle, sterol-auxotrophic oomycetes very likely require sterols, and these have to be recruited. Plant pathogens feel comfortable when growing in close intimacy with their hosts, and that is also the niche where they form propagules for dispersal and resting spores for survival. It is therefore conceivable that plant pathogenic oomycetes exploit sterols from their hosts for growth and reproduction.

That sterols are essential for oomycetes is further demonstrated by the strong growth inhibition of sterol-prototroph Saprolegniales by SBIs, fungicides interfering with sterol biosynthesis [13,14]. Similarly, sterol sequestration inhibits growth of *Phytophthora*. This was nicely demonstrated by Gamir and colleagues [18] who revealed the mode of action of the pathogenesis-related protein PR-1, one of the first described markers of plant immune signaling. Nearly 5 decades after its first discovery as the most abundant extracellular protein in pathogen-challenged tobacco plants, PR-1 turned out to have sterol-binding activity. It is a member of the cysteine-rich secretory protein, antigen 5, and pathogenesis-related-1 (CAP) superfamily that share a 150 amino acid CAP domain [18,19]. The fact that sterol-binding activity of PR-1 is pivotal for inhibiting the development of *Phytophthora brassicae* [18], presumably by acting as competitor of sterol sensors or sterol transport proteins or by sequestering sterols from the membrane, could explain its anti-oomycete activity observed in many previous studies [19].

Sterols is a collective term for organic compounds composed of 4 rings and variable site chains and bonds. In *Phytophthora*, this variation in structure correlates with diverse activities [10,15,20]. For example, *Phytophthora cactorum* seems to have a preference for taking up Δ^5^ sterols over Δ^5,7^ sterols and tends to transform Δ^5,7^ sterols into Δ^5^ ones [21], probably mediated by DHCR7. This is corroborated by our recent study, which showed that DHCR7 in *P*. *capsici* is indeed responsible for reducing the double bond at that position [17]. When comparing different sterols or sterol mixtures, some stand out in showing higher activities than others. In view of host–pathogen coevolution, the variation in effects of the various sterols and the preference of different *Phytophthora* species for certain sterols is not surprising. Plants each have their own cocktail of sterols, varying in composition and amounts in different plant organs, and this is the sterol diet offered to pathogens.

### Are oomycete plant pathogens dependent on plant sterols?

In the arms race between plants and pathogens, plants take advantage of their innate immune system to efficiently detect and ward off enemies. Upon recognition of pathogen-derived microbe-associated molecular patterns (MAMPs), plants activate their defense machinery and without counter-defense, the invasion is blocked. Also, sterols take part in this arms race: Ergosterol is a MAMP and likely present in the cocktail of MAMPs that triggers defense against fungi [22]. Moreover, fungi are harmed by saponins, secondary plant metabolites that compromise plasma membrane integrity by interacting with 3β-hydroxyl sterols, e.g., avenacin in oat and α-tomatine in tomato. Their fungicidal activity is counteracted by pathogens producing saponin-degrading enzymes as virulence factors resulting in disease [4]. Unlike fungi, sterol auxotrophs are not affected by saponins, in line with their sterol-independent growth. It is more likely that they exploit host sterols to facilitate their own development. With that in mind, manipulating supply of sterols might be a smart strategy to fend off oomycete pathogens. This can be accomplished by competing for sterols as demonstrated for PR-1 [18]. Upon pathogen attack, PR-1 is secreted into the apoplast, but its final destination is not known. In fact, in vitro assays showed that uptake of PR-1 by *P*. *brassicae* is required for inhibiting growth. This points to some kind of intracellular competition, presumably with transporters or sensors involved in sterol metabolism or signaling. Alternatively, sterol supply in plants could be modified in such a way that the cocktail of sterols is less attractive for oomycetes.

Studies testing the hypothesis that sterols are determinants of resistance are rare. Attempts to find correlations between sterol content in potato cultivars and level of resistance to the late blight pathogen *Phytophthora infestans* were inconclusive [9,23]. This is not surprising given the variations in relative amounts of major sterol constituents among potato cultivars and the notion that sterol composition not only varies during plant development but is also influenced by growth conditions and biotic stress. An example showing that modifying sterol composition can cause gain of disease resistance is the finding that *Arabidopsis* mutants lacking the capacity to synthesize a C22 desaturase (i.e., cytochrome P450 CYP710A1), and, hence, to convert β-sitosterol into stigmasterol, are hardly susceptible to the bacterium *Pseudomonas syringae*. In wild-type plants, *CYP710A1* expression is induced by pathogens, including *P*. *infestans*, and MAMP treatment. This leads to production of stigmasterol thereby increasing the stigmasterol:β-sitosterol ratio in membranes, and promoting susceptibility to *Ps*. *syringae* [24]. Whether stigmasterol in *Arabidopsis* is a determining factor for susceptibility to oomycete pathogens remains to be tested. Another defense-related enzyme is PSAT1, a phospholipid:sterol acyltransferase catalyzing formation of sterol esters (i.e., conjugates of sterols and fatty acids) and affecting sterol homeostasis [25]. In *Arabidopsis*, *P*. *infestans* infection leads to 2-fold higher levels of sterol esters. In PSAT1 mutants, this level is decreased, while sterol glycoside levels are increased. *P*. *infestans* cannot infect *Arabidopsis*. The nonhost resistance is manifested by a local cell death response and efficient callose deposition to block entry of the pathogen. While PSAT1 mutants are not compromised in resistance, callose deposition is deregulated, and cell death is more spread [25]. This points to a rewiring of signaling pathways due to altered sterol homeostasis.

### How do oomycetes recruit sterols?

For sterol auxotrophs, sterol recruitment is essential. In insects that recruit sterols from the gut lumen, several receptors and transport proteins regulating the flow of sterols into and within cells have been identified [5]. In oomycetes, however, the sterol recruitment process is largely unknown. Proteins that are thought to act as sterol snatchers are elicitins, secreted proteins sharing a highly conserved 98-amino acid domain that forms a hydrophobic cavity [26,27]. Of the multiple elicitin (ELI) and elicitin-like (ELL) proteins encoded in *Phytophthora* genomes, only the clade-1 ELIs (ELI-1) have been intensively studied, already since the 1980s. The ELI-1 cryptogein secreted by *Phytophthora cryptogea* was discovered because of its ability to elicit necrosis in tobacco [28]. In retrospect, it is one of the first identified MAMPs. In 2015, Du and colleagues [29] identified a plant receptor that mediates recognition of the canonical ELI domain and confers enhanced resistance to *P*. *infestans* when ectopically expressed in potato.

Because of the structural resemblance of the elicitin domain with nonspecific lipid-transfer proteins (nsLTPs) and its high-affinity binding to sterols, ELIs were proposed to serve as sterol carriers [26,30,31]. Mutated versions of ELI-1 that fail to bind sterols are still active as MAMP, implying that these 2 activities are independent [32]. In contrast to ELI-1, all other ELIs (ELI-2, ELI-3, and ELI-4) have repeat-rich carboxyl-terminal extensions with features reminiscent of cell wall proteins. Possibly, they serve as anchors like sticks of lollipops holding the elicitin domain attached to hyphae while snatching sterols from the environment. ELLs also have carboxyl-terminal extensions, but their elicitin-like domains are more variable and lack necrosis-inducing activity. It is unknown whether ELLs bind sterols; their structure and potential function remain to be investigated.

ELIs and ELLs are oomycete-specific proteins, but, strikingly, ELIs are exclusively found in the sterol-auxotrophic *Phytophthora* and *Pythium* species [27]. This is in line with the hypothesis that plant pathogenic oomycetes exploit ELIs to recruit sterols from their hosts, while those pathogenic on animals or with high saprophytic capability are sterol prototrophs having their own sterol supply. As yet, it is not clear how sterols, once trapped by elicitins, are taken up and if there are still other ways to recruit sterols provided by the host.

### How do oomycetes sense sterols?

The observation that sterols promote vegetative growth and reproduction in *Phytophthora* implies that these organisms can sense sterols and possess intracellular signaling networks triggered by sterols. This, in turn, relies on a balanced system of intracellular sterol transport and distribution, sterol storage and release, and also sterol sensing, a system that is likely supported by sterol-binding and sterol-sensing proteins and enzymes for sterol biosynthesis or transforming free sterols into sterol conjugates. Although intensively studied in humans and model organisms such as yeast, insight in these processes is still rudimentary. Genome mining predicts that oomycetes possess homologs of proteins known to be involved in sterol homeostasis and metabolism in other organisms. For instance, *P*. *capsici* and *Phytophthora sojae* each have at least 4 genes encoding proteins with a sterol-sensing domain (SSD), and, likely, these are conserved throughout the genus and beyond. A recent study highlighted a homolog as a putative candidate for a mating-hormone receptor in the downy mildew *Plasmopara viticola*. As yet, this is solely based on co-localization in a 570-kb region containing 40 genes and awaits further verification [33]. Proteins containing an SSD domain are known to play a role in sterol absorption or transportation, and, intriguingly, also in several signal transduction pathways [5,34–36]. The role of the SSD containing proteins in *Phytophthora* is not known, but more in-depth studies might reveal whether and how they participate in signaling in sterol auxotrophs.

Untangling signaling pathways in oomycetes is challenging especially because they have many unique proteins, such as novel potential phospholipid-modifying enzymes [37] and peculiar G protein–coupled receptors (GPCRs) [38]. Phospholipids and sterols occupy similar niches, and GPCRs are often key players in sterol signaling networks. Uncovering the mysterious route of sterols in oomycetes should reveal how the various components interact and how sterol auxotroph oomycetes manage to recruit and exploit sterols for survival in their natural habitat. Equally intriguing is to find out how interfering with sterol-based processes provides leads for novel disease control strategies.

## References

[1] Dufourc EJ. Sterols and membrane dynamics. J Chem Biol. 2008;1(1–4):63–77. doi: 10.1007/s12154-008-0010-6 19568799 PMC2698314

[2] Platt FM, Wassif C, Colaco A, Dardis A, Lloyd-Evans E, Bembi B, et al. Disorders of cholesterol metabolism and their unanticipated convergent mechanisms of disease. Annu Rev Genomics Hum Genet. 2014;15(1):173–94. doi: 10.1146/annurev-genom-091212-153412 25184529 PMC6292211

[3] Lepesheva GI, Friggeri L, Waterman MR. Cyp51 as drug targets for fungi and protozoan parasites: past, present and future. Parasitology. 2018;145(14):1820–36. doi: 10.1017/S0031182018000562 29642960 PMC6185833

[4] Kazan K, Gardiner DM. Targeting pathogen sterols: Defence and counterdefence? PLoS Pathog. 2017;13(5):e1006297. doi: 10.1371/journal.ppat.1006297 28542599 PMC5436867

[5] Jing X, Behmer ST. Insect sterol nutrition: physiological mechanisms, ecology, and applications. Annu Rev Entomol. 2020;65:251–71. doi: 10.1146/annurev-ento-011019-025017 31600456

[6] Shamsuzzama LR, Trabelcy B, Langier Goncalves I, Gerchman Y, Sapir A. Metabolic reconfiguration in *C*. *elegans* suggests a pathway for widespread sterol auxotrophy in the animal kingdom. Curr Biol. 2020;30(15):3031–8. doi: 10.1016/j.cub.2020.05.070 32559444

[7] Keeling PJ, Burki F. Progress towards the tree of eukaryotes. Curr Biol. 2019;29(16):808–17. doi: 10.1016/j.cub.2019.07.031 31430481

[8] Kamoun S, Furzer O, Jones JDG, Judelson HS, Ali GS, Dalio RJD, et al. The top 10 oomycete pathogens in molecular plant pathology. Mol Plant Pathol. 2015;16(4):413–34. doi: 10.1111/mpp.12190 25178392 PMC6638381

[9] Langcake P. Sterols in potato leaves and their effects on growth and sporulation of *Phytophthora infestans*. Mycol Res. 1974;63(3):573–86.

[10] Marshall JA, Dennis AL, Kumazawa T, Haynes AM, Nes WD. Soybean sterol composition and utilization by *Phytophthora sojae*. Phytochemistry. 2001;58(3):423–8. doi: 10.1016/s0031-9422(01)00219-9 11557074

[11] Nes WD. Biosynthesis of cholesterol and other sterols. Chem Rev. 2011;111(10):6423–51. doi: 10.1021/cr200021m 21902244 PMC3191736

[12] Desmond E, Gribaldo S. Phylogenomics of sterol synthesis: insights into the origin, evolution, and diversity of a key eukaryotic feature. Genome Biol Evol. 2009;1:364–81. doi: 10.1093/gbe/evp036 20333205 PMC2817430

[13] Madoui MA, Bertrand-Michel J, Gaulin E, Dumas B. Sterol metabolism in the oomycete *Aphanomyces euteiches*, a legume root pathogen. New Phytol. 2009;183(2):291–300. doi: 10.1111/j.1469-8137.2009.02895.x 19496952

[14] Warrilow AG, Hull CM, Rolley NJ, Parker JE, Nes WD, Smith SN, et al. Clotrimazole as a potent agent for treating the oomycete fish pathogen *Saprolegnia parasitica* through inhibition of sterol 14α-demethylase (CYP51). Appl Environ Microbiol. 2014;80(19):6154–66. doi: 10.1128/AEM.01195-14 25085484 PMC4178690

[15] Hendrix JW. Sterols in growth and reproduction of fungi. Annu Rev Phytopathol. 1970;8(1):111–30.

[16] Dahlin P, Srivastava V, Ekengren S, McKee LS, Bulone V. Comparative analysis of sterol acquisition in the oomycetes *Saprolegnia parasitica* and *Phytophthora infestans*. PLoS ONE. 2017;12(2):e0170873. doi: 10.1371/journal.pone.0170873 28152045 PMC5289490

[17] Wang W, Zhang F, Zhang S, Xue Z, Xie L, Govers F, et al. *Phytophthora capsici* sterol reductase PcDHCR7 has a role in mycelium development and pathogenicity. bioRxiv. doi: 10.1101/2021.04.17.440084 PMC8984297 35382565

[18] Gamir J, Darwiche R, Van’t Hof P, Choudhary V, Stumpe M, Schneiter R, et al. The sterol-binding activity of pathogenesis-related protein 1 reveals the mode of action of an antimicrobial protein. Plant J. 2017;89(3):502–9. doi: 10.1111/tpj.13398 27747953

[19] Breen S, Williams SJ, Outram M, Kobe B, Solomon PS. Emerging insights into the functions of pathogenesis-related protein 1. Trends Plant Sci. 2017;22(10):871–9. doi: 10.1016/j.tplants.2017.06.013 28743380

[20] Nes WD, Saunders GA, Heftmann E. Role of steroids and triterpenoids in the growth and reproduction of *Phytophthora cactorum*. Lipids. 1982;17(3):178–83.

[21] Knights BA, Elliott CG. Metabolism of Δ7 and Δ 5,7 sterols by *Phytophthora cactorum*. Biochim Biophys Acta. 1976;441(2):341–6. doi: 10.1016/0005-2760(76)90178-8 952995

[22] Klemptner RL, Sherwood JS, Tugizimana F, Dubery IA, Piater LA. Ergosterol, an orphan fungal microbe-associated molecular pattern (MAMP). Mol Plant Pathol. 2014;15(7):747–61. doi: 10.1111/mpp.12127 24528492 PMC6638689

[23] Elliott CG, Knights BA. Interactions between steroids in the growth of *Phytophthora*. J Sci Food Agric. 1969;20(7):406–8.

[24] Griebel T, Zeier J. A role for β-sitosterol to stigmasterol conversion in plant-pathogen interactions. Plant J. 2010;63(2):254–68. doi: 10.1111/j.1365-313X.2010.04235.x 20444228

[25] Kopischke M, Westphal L, Schneeberger K, Clark R, Ossowski S, Wewer V, et al. Impaired sterol ester synthesis alters the response of *Arabidopsis thaliana* to *Phytophthora infestans*. Plant J. 2013;73(3):456–68. doi: 10.1111/tpj.12046 23072470

[26] Blein J-P. Coutos-Thévenot Pierre, Marion D, Ponchet M. From elicitins to lipid-transfer proteins: a new insight in cell signalling involved in plant defence mechanisms. Trends Plant Sci. 2002;7(7):293–6. doi: 10.1016/s1360-1385(02)02284-7 12119165

[27] Jiang RHY, Tyler BM, Whisson SC, Hardham AR, Govers F. Ancient origin of elicitin gene clusters in *Phytophthora* genomes. Mol Biol Evol. 2006;23:338–51. doi: 10.1093/molbev/msj039 16237208

[28] Ricci P, Bonnet P, Huet JC, Sallantin M, Beauvais-Cante F, Bruneteau M, et al. Structure and activity of proteins from pathogenic fungi *Phytophthora* eliciting necrosis and acquired resistance in tobacco. Eur J Biochem. 1989;183(3):555–63. doi: 10.1111/j.1432-1033.1989.tb21084.x 2776750

[29] Du J, Verzaux E, Chaparro-Garcia A, Bijsterbosch G, Keizer LP, Zhou J, et al. Elicitin recognition confers enhanced resistance to *Phytophthora infestans* in potato. Nat Plants. 2015;1(4):15034. doi: 10.1038/nplants.2015.34 27247034

[30] Mikes V, Milat ML, Ponchet M, Ricci P, Blein JP. The fungal elicitor cryptogein is a sterol carrier protein. FEBS Lett. 1997;416(2):190–2. doi: 10.1016/s0014-5793(97)01193-9 9369212

[31] Vauthrin S, Mikes V, Milat ML, Ponchet M, Maume B, Osman H, et al. Elicitins trap and transfer sterols from micelles, liposomes and plant plasma membranes. Biochim Biophys Acta Biomembr. 1999;1419(2):335–42. doi: 10.1016/s0005-2736(99)00083-8 10407084

[32] Dokládal L, Obořil M, Stejskal K, Zdráhal Z, Ptáčková N, Chaloupková R, et al. Physiological and proteomic approaches to evaluate the role of sterol binding in elicitin-induced resistance. J Exp Bot. 2012;63(5):2203–15. doi: 10.1093/jxb/err427 22223811 PMC3295402

[33] Dussert Y, Legrand L, Mazet ID, Couture C, Piron MC, Serre RF, et al. Identification of the first oomycete mating-type locus sequence in the grapevine downy mildew pathogen, *Plasmopara viticola*. Curr Biol. 2020;30(20):3897–907. doi: 10.1016/j.cub.2020.07.057 32795448 PMC7116238

[34] Kuwabara PE, Labouesse M. The sterol-sensing domain: multiple families, a unique role? Trends Genet. 2002;18(4):193–201. doi: 10.1016/s0168-9525(02)02640-9 11932020

[35] Altmann SW, Davis HR Jr, Zhu LJ, Yao X, Hoos LM, Tetzloff G, et al. Niemann-Pick C1 Like 1 protein is critical for intestinal cholesterol absorption. Science. 2004;303(5661):1201–1204. doi: 10.1126/science.1093131 14976318

[36] Jia L, Betters JL, Yu L. Niemann-Pick C1-Like 1 (NPC1L1) protein in intestinal and hepatic cholesterol transport. Annu Rev Physiol. 2011;73(1):239–59. doi: 10.1146/annurev-physiol-012110-142233 20809793 PMC3965667

[37] Meijer HJ, Govers F. Genomewide analysis of phospholipid signaling genes in *Phytophthora* spp.: novelties and a missing link. Mol Plant Microbe Interact. 2006;19(12):1337–47. doi: 10.1094/MPMI-19-1337 17153918

[38] Hoogen van den J, Govers F. GPCR-bigrams: enigmatic signaling components in oomycetes. PLoS Pathog. 2018;14(7):e1007064. doi: 10.1371/journal.ppat.1007064 29975773 PMC6033456

